# Supplementary material for: A genome-wide atlas of antibiotic susceptibility targets and pathways to tolerance
Source: Nat Commun. 2022 Jun 7;13:3165. doi: 10.1038/s41467-022-30967-4 (PMC9174251; doi:10.1038/s41467-022-30967-4)
Supplement: Supplementary file 1 — Supplementary Information [file 41467_2022_30967_MOESM1_ESM.pdf]

SUPPLEMENTAL FIGURES

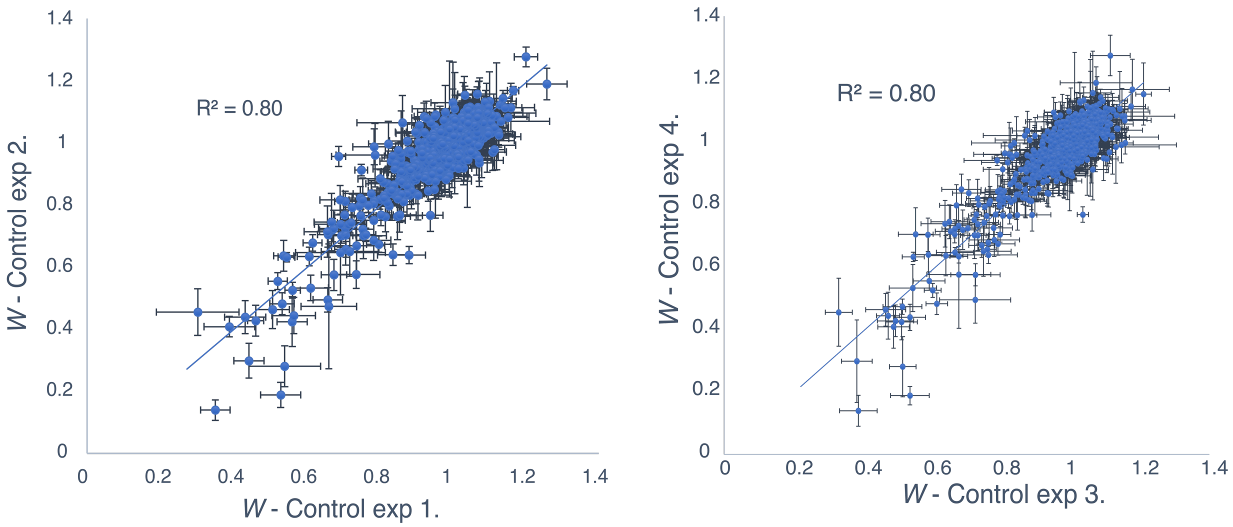

Supplemental Figure 1. Tn-Seq experiment correlations. Comparing fitness (*W*) among four Tn-Seq experiments, highlighting reproducibility among experiments, which is similar to previous projects[1-10]. Source data are provided as a Source Data file.

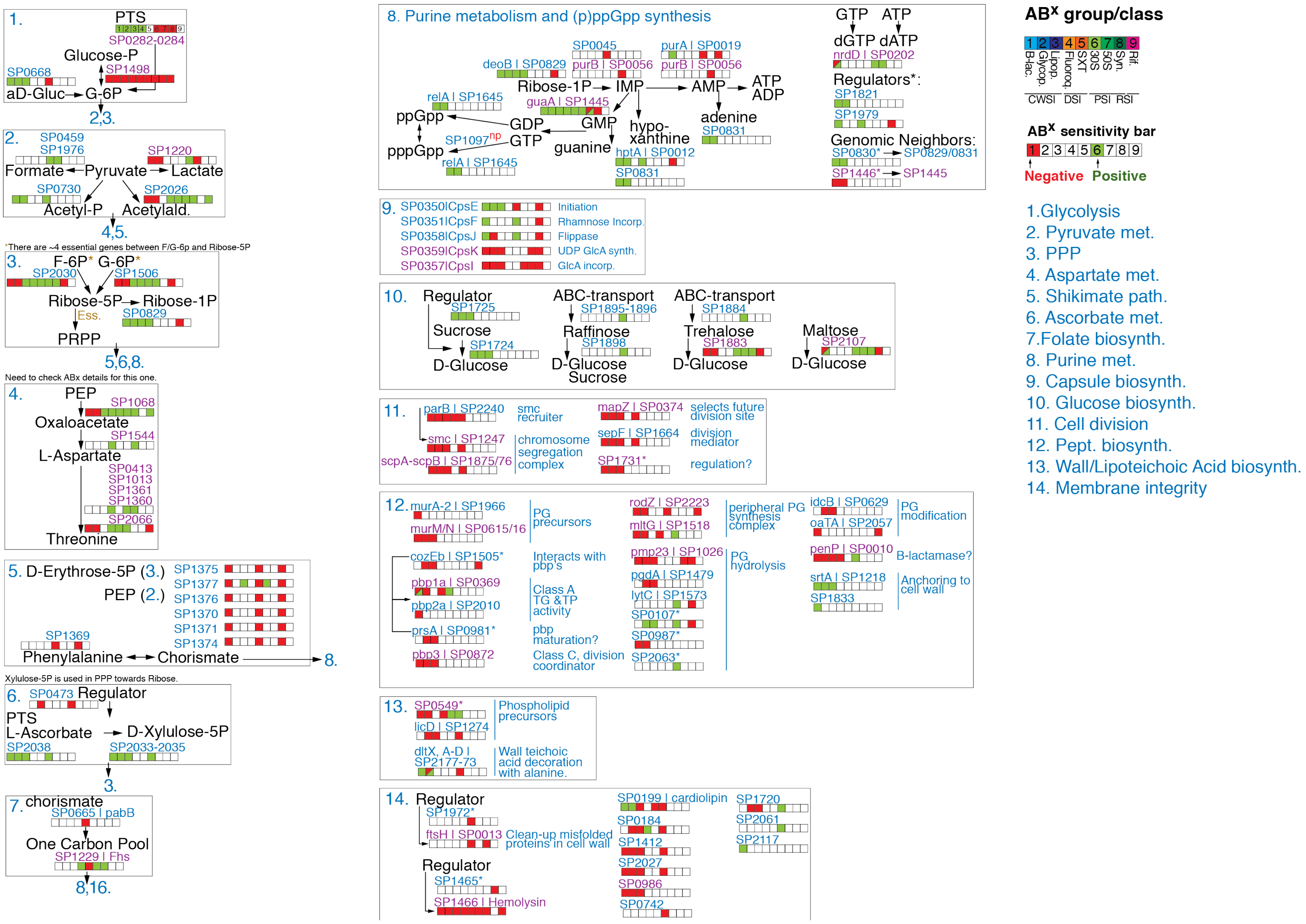

Supplemental Figure 2. Detailed view of groups/processes 1-14, highlighting how modulation of specific targets within each process leads to changes in antibiotic sensitivity. Where possible, genes are ordered according to their place in a process/pathway, and gene numbers (SP_) are combined with gene names and annotation. Each indicated gene is combined with an ‘antibiotic sensitivity bar’ indicating whether disruption leads to increased (red/negative fitness) or decreased (green/positive fitness) sensitivity to a specific or group of antibiotics. When phenotypic responses are the same,

multiple genes are indicated with a single bar (e.g. SP0282/SP0283/SP0284 in glycolysis, or SP0413/SP1013/SP1361/SP1360 in Aspartate metabolism). Gene numbers in blue have no effect on growth in the absence of antibiotics when knocked out, while gene numbers in purple have a significant growth defect in the absence of ABXs (see for detailed fitness in the absence and presence of antibiotics Supplementary Data 2). Essential genes are not indicated and genes with an asterisk have a partial or tentative annotation that has not been resolved.

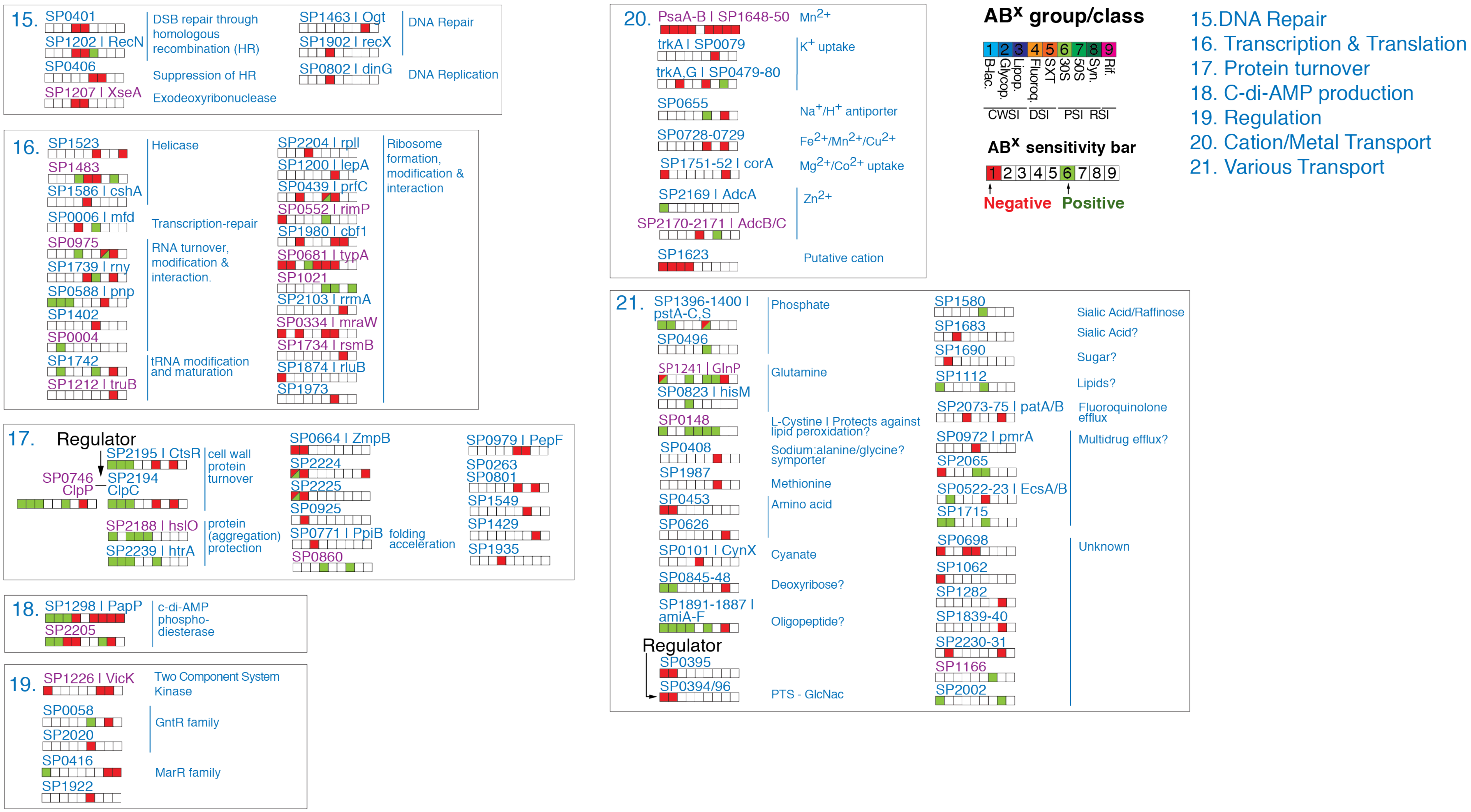

Supplemental Figure 3. Detailed view of groups/processes 15-21, highlighting how modulation of specific targets within each process leads to changes in antibiotic sensitivity. Coloring and layout is the same as Supplemental Figure 2 and Figure 3c in the main text.

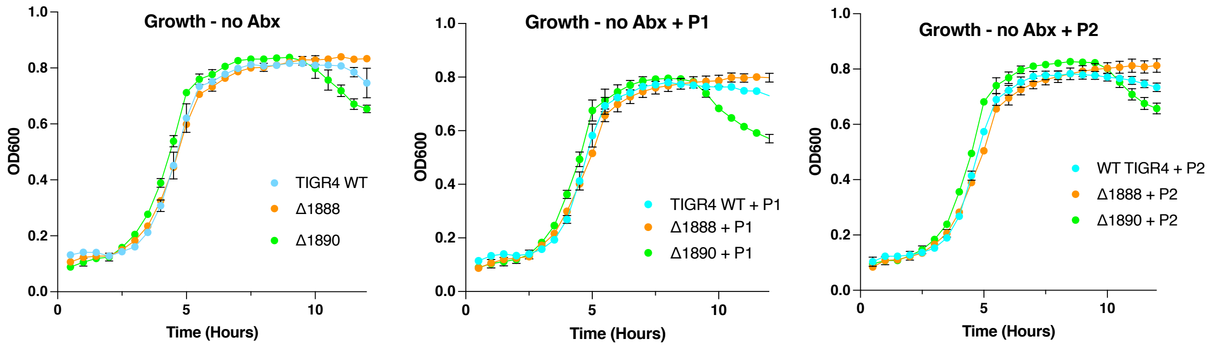

Supplemental Figure 4. Growth Curves of WT, and deletion mutants Δ*amiE* (SP_1888) and Δ*amiC* (SP_1890) in rich media without antibiotics and supplemented with 5mM peptide P1 or P2. Mean values +/- SEM are shown from n=3 independent experiments. Source data are provided as a Source Data file.

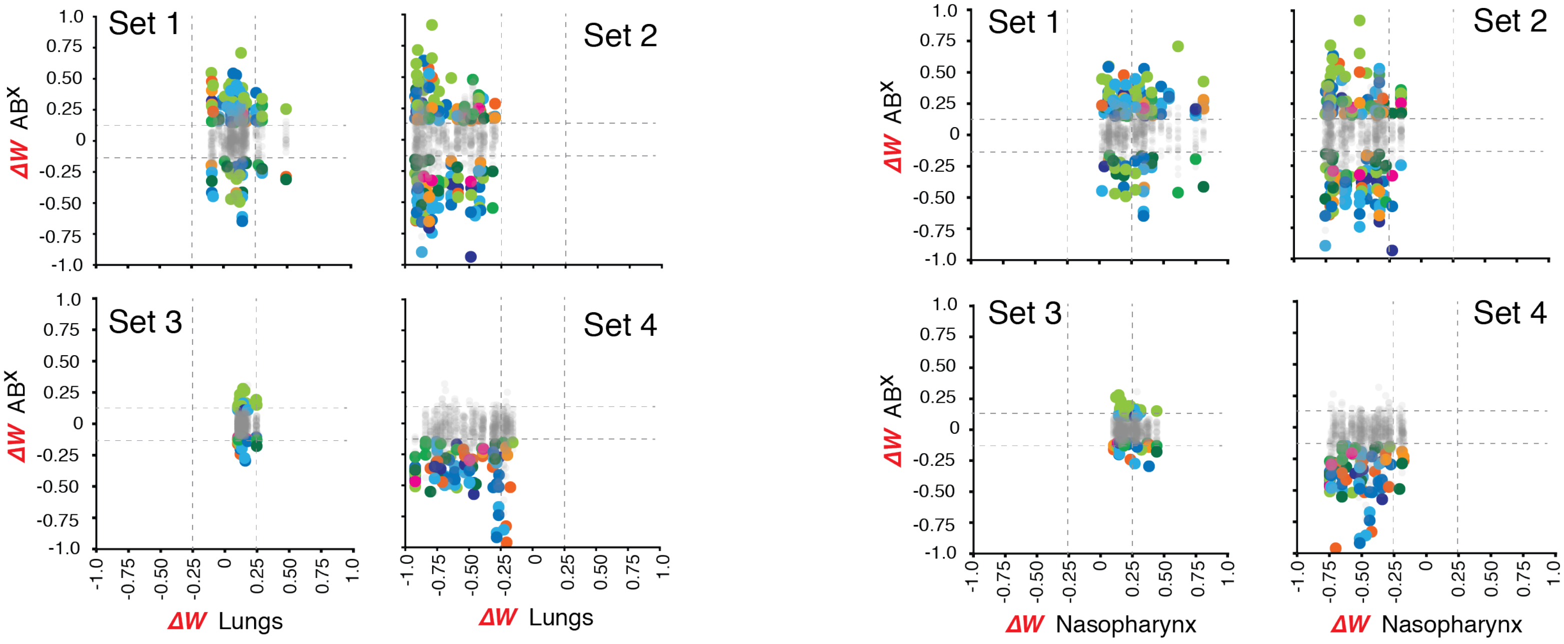

Supplemental Figure 5. Set distribution Lung and Nasopharynx. Based on *in vivo* and ABX Tn-Seq data, four gene-sets consisting of 34 genes each were compiled with specific fitness profiles in the presence of antibiotics and *in vivo.* Shown are the *in vivo* effects for lung (left) and nasopharynx (right). *ΔW* represents the fitness difference of a gene in a specific condition (e.g., an antibiotic, *in vivo)* minus its fitness *in vitro* in rich medium. Dashed lines indicate significance cut-offs, greyed-out dots indicate genes with no significant change in fitness in the presence of antibiotics, colors represent antibiotics and are the same as in Fig. 1.

Supplementary References

1 van Opijnen, T., Bodi, K. L. & Camilli, A. Tn-seq: high-throughput parallel sequencing for fitness and genetic interaction studies in microorganisms. *Nat Methods* **6**, 767-772, doi:10.1038/nmeth.1377 (2009).

2 van Opijnen, T. & Camilli, A. A fine scale phenotype-genotype virulence map of a bacterial pathogen. *Genome Res* **22**, 2541-2551, doi:10.1101/gr.137430.112 (2012).

3 Mann, B. *et al.* Control of virulence by small RNAs in Streptococcus pneumoniae. *PLoS pathogens* **8**, e1002788, doi:10.1371/journal.ppat.1002788 (2012).

4 van Opijnen, T. & Camilli, A. Transposon insertion sequencing: a new tool for systems-level analysis of microorganisms. *Nature reviews. Microbiology* **11**, 435-442, doi:10.1038/nrmicro3033 (2013).

5 Carter, R. *et al.* Genomic Analyses of Pneumococci from Children with Sickle Cell Disease Expose Host-Specific Bacterial Adaptations and Deficits in Current Interventions. *Cell Host and Microbe* **15**, 587-599, doi:papers://58864D70-D09B-4A16-B641-1A3EEB7FE0BA/Paper/p22252 (2014).

6 van Opijnen, T., Dedrick, S. & Bento, J. Strain Dependent Genetic Networks for Antibiotic-Sensitivity in a Bacterial Pathogen with a Large Pan-Genome. *PLoS pathogens* **12**, e1005869, doi:10.1371/journal.ppat.1005869 (2016).

7 Jensen, P. A., Zhu, Z. & van Opijnen, T. Antibiotics Disrupt Coordination between Transcriptional and Phenotypic Stress Responses in Pathogenic Bacteria. *Cell Rep* **20**, 1705-1716, doi:10.1016/j.celrep.2017.07.062 (2017).

8 Thibault, D. *et al.* Droplet Tn-Seq combines microfluidics with Tn-Seq for identifying complex single-cell phenotypes. *Nat Commun* **10**, 5729, doi:10.1038/s41467-019-13719-9 (2019).

9 Geisinger, E. *et al.* Antibiotic hypersensitivity signatures identify targets for attack in the *Acinetobacter baumannii* cell envelope. *Nature Communications in press* (2020).

10 van Opijnen, T. & Levin, H. L. Transposon Insertion Sequencing, a Global Measure of Gene Function. *Annu Rev Genet*, doi:10.1146/annurev-genet-112618-043838 (2020).
