# Supplementary material for: A genome-wide atlas of antibiotic susceptibility targets and pathways to tolerance
Source: Nat Commun. 2022 Jun 7;13:3165. doi: 10.1038/s41467-022-30967-4 (PMC9174251; doi:10.1038/s41467-022-30967-4)
Supplement: Supplementary file 3 — Description of Additional Supplementary Files [file 41467_2022_30967_MOESM3_ESM.pdf]

Description of Additional Supplementary Files

Supplemental Data 1. Antibiotics, concentrations, abbreviations and minimum inhibitory concentrations (MICs). Twenty antibiotics are used in this study at concentrations that reduce growth by 30-50%. Moreover, MICs for TIGR4 and derived mutants are listed.

Supplemental Data 2. Tn-Seq data from *in vitro* and *in vivo* experiments. Tn-Seq was performed *in vitro* in the presence of 20 antibiotics and *in vivo* in lung and the nasopharynx.

Supplemental Data 3. Gene x Condition matrix. A gene x condition matrix based on 20 antibiotic conditions from experiments performed here, supplemented with 17 conditions consisting of Sucrose, Fructose, Cellobiose, Raffinose, Sialic Acid, Galactose, Mannose, Maltose, GlcNac, Bipyridyl, transformation, hydrogen-peroxide, methyl-methane sulfonate, pH6, temperature, Norfloxacin. Genes with missing data were removed resulting in a 1519 gene x 37 condition matrix.

Supplemental Data 4. Correlation matrix. Genes and conditions were correlated using a Pearson's correlation coefficient and a Spearman's correlation coefficient. Resulting in two 1519x1519, gene vs gene matrices.

Supplemental Data 5. Stability matrix. A stability test was performed to determine the robustness and quality of each edge in the network by building a correlation matrix from partial data. 30 conditions were selected 100 times to build a correlation matrix and using the same cutoff criteria a co-fitness matrix was compiled. Every edge with a correlation value above the threshold was assigned a 1 and every edge below the cut-off 0. This resulted in 100 binary matrices which were then summated, resulting in every gene vs gene interaction being assigned a stability score with a value N out of 100.

Supplemental Data 6. Network Edge Table. A significance cutoff was applied to the 1519x1519, gene vs gene matrices and correlations ≥0.75 were retained and used as edges to build a co-fitness network consisting of 1519 genes and 2399 edges. An edge-weighted spring embedded layout was applied with Cytoscape, with the absolute correlation value as the edge weight. This results in a network with several major clusters and multiple genes unconnected to the main network.

Supplemental Data 7. SAFE analysis. A SAFE (Spatial Analysis of Functional Enrichment) analysis on the co-fitness network was performed with Cytoscape. Attributes were assigned by merging KEGG pathway annotation and available functional category annotations, which covers 94% of the genes within the network. The distance threshold was set to the 1st percentile of the map-weighted distance, the Jaccard similarity index was set to 0.5, and nodes in different landscapes were retained.

Supplemental Data 8. Growth and Tolerance data. Growth and tolerance data for TIGR4 and derived mutants used in this study.

Supplemental Data 9. Purine metabolism. LC/MS data in the absence and presence of mupirocin for ppGpp, pppGpp, GTP, GDP, ATP and ADP.

Supplemental Data 10. Competitive indexes. Competitive index (C.I.) data for SP_0829 and SP_1396 measured *in vivo* in the nasopharynx and lung in the absence and presence of antibiotics.
