# Supplementary material for: A genome-wide atlas of antibiotic susceptibility targets and pathways to tolerance
Source: Nat Commun. 2022 Jun 7;13:3165. doi: 10.1038/s41467-022-30967-4 (PMC9174251; doi:10.1038/s41467-022-30967-4)
Supplement: Supplementary file 14 — Reporting Summary [file 41467_2022_30967_MOESM14_ESM.pdf]

nature portfolio

Corresponding author(s): Tim van Opijnen

Last updated by author(s): 2022 04 22

Reporting Summary

Nature Portfolio wishes to improve the reproducibility of the work that we publish. This form provides structure for consistency and transparency in reporting. For further information on Nature Portfolio policies, see our Editorial Policies and the Editorial Policy Checklist.

Statistics

For all statistical analyses, confirm that the following items are present in the figure legend, table legend, main text, or Methods section.

n/a	Confirmed	
☐	☒	The exact sample size (*n*) for each experimental group/condition, given as a discrete number and unit of measurement
☐	☒	A statement on whether measurements were taken from distinct samples or whether the same sample was measured repeatedly
☐	☒	The statistical test(s) used AND whether they are one- or two-sided<br>*Only common tests should be described solely by name; describe more complex techniques in the Methods section.*
☐	☒	A description of all covariates tested
☐	☒	A description of any assumptions or corrections, such as tests of normality and adjustment for multiple comparisons
☐	☒	A full description of the statistical parameters including central tendency (e.g. means) or other basic estimates (e.g. regression coefficient) AND variation (e.g. standard deviation) or associated estimates of uncertainty (e.g. confidence intervals)
☐	☒	For null hypothesis testing, the test statistic (e.g. *F*, *t*, *r*) with confidence intervals, effect sizes, degrees of freedom and *P* value noted<br>*Give P values as exact values whenever suitable.*
☒	☐	For Bayesian analysis, information on the choice of priors and Markov chain Monte Carlo settings
☒	☐	For hierarchical and complex designs, identification of the appropriate level for tests and full reporting of outcomes
☐	☒	Estimates of effect sizes (e.g. Cohen's *d*, Pearson's *r*), indicating how they were calculated

Our web collection on statistics for biologists contains articles on many of the points above.

Software and code

Policy information about availability of computer code

Data collection	Software and code used for data collection include Aerobio v2.3, Analyst software v1.7
Data analysis	Software used for data analysis include Cytoscape (v3.5.1), Prismv9, Waters MassLynx v4.2, MultiQuantv3.0.2, Aerobio v2.3.

For manuscripts utilizing custom algorithms or software that are central to the research but not yet described in published literature, software must be made available to editors and reviewers. We strongly encourage code deposition in a community repository (e.g. GitHub). See the Nature Portfolio guidelines for submitting code & software for further information.

Data

Policy information about availability of data

All manuscripts must include a data availability statement. This statement should provide the following information, where applicable:

- Accession codes, unique identifiers, or web links for publicly available datasets
- A description of any restrictions on data availability
- For clinical datasets or third party data, please ensure that the statement adheres to our policy

The datasets generated during and/or analysed during the current study are available in the SRA BioProject accession number PRJNA750080

Field-specific reporting

Life sciences study design

All studies must disclose on these points even when the disclosure is negative.

Sample size	Sample size represents the number of biological replicates in an experiment in our study. Sample sizes are 6 for Tn-Seq, 7 or more for mouse experiments, 3 or more for growth assays and all other experiments. Sample sizes are detailed in manuscript.
Data exclusions	No data/replicates were excluded from analyses.
Replication	Tn-Seq were successfully replicated in 6 biological replicates. Mouse experiments, growth assays and all other experiments were succesfully replicated at least three times.
Randomization	Randomization by permutation testing was performed as part of the network analyses.
Blinding	Experimenters were blinded to the sample identities during experimentation, sample preparation and analyses for all experiments.

Reporting for specific materials, systems and methods

We require information from authors about some types of materials, experimental systems and methods used in many studies. Here, indicate whether each material, system or method listed is relevant to your study. If you are not sure if a list item applies to your research, read the appropriate section before selecting a response.

Materials & experimental systems

n/a	Involved in the study	
☐	☒	Antibodies
☒	☐	Eukaryotic cell lines
☒	☐	Palaeontology and archaeology
☐	☒	Animals and other organisms
☒	☐	Human research participants
☒	☐	Clinical data
☒	☐	Dual use research of concern

Methods

n/a	Involved in the study	
☒	☐	ChIP-seq
☒	☐	Flow cytometry
☒	☐	MRI-based neuroimaging

Antibodies

Antibodies used	a-SP1505 (Genscript custom order), goat anti-rabbit IgG-HRP (Bio-Rad catalog #1721019), mouse-anti-His-antibody, LIFE Technologies (catalog #37-2900), Goat Anti-Mouse IgG (H + L)-HRP Conjugate (Biorad, catalog# 1706516)
Validation	Lab confirmation of the SP_1505 expression construct was undertaken using a 1:3000 dilution of mouse-anti-His-antibody, sourced from LIFE Technologies (catalog #37-2900) followed by Goat Anti-Mouse IgG (H + L)-HRP Conjugate (Biorad, catalog# 1706516), used at 1:5000 dilution. a-SP1505 were custom generated by Gesncript, antibodies were tested on nitrocellulose membranes, which were blocked overnight in 5% NFDM and treated with primary antibody against SP_1505 at a concentration of 1:500. After washing, membranes were treated with secondary antibody goat anti-rabbit IgG-HRP (Bio-Rad catalog #1721019) at a concentration of 1:3000. a-SP_1505 were further tested through in vitro killing assays and in vivo survival assays.

Animals and other organisms

Policy information about studies involving animals; ARRIVE guidelines recommended for reporting animal research

Laboratory animals	Mus musculus, Swiss Webster ~7wks old obtained from Taconic, Inc. Balb/c, ~7wks old obtained from Jackson Labs. Experiments approved by the Boston College IACUC under protocol 2019-007-01 to Dr. Tim van Opijnen, PhD, and approved under St. Jude Children's Research Hospital IACUC approved protocol #538 to Dr. Jason Rosch. Mice were housed with a 12hr/12hr:dark/light cycle. The room temperature set point was 71 degrees F (+/- 2 degrees) and the humidity setpoint was 40%.
Wild animals	none
Field-collected samples	none
Ethics oversight	Research presented within the manuscript complies with all relevant ethical regulations and protocols as approved by the Boston College environmental health and safety board. Mouse experiments approved by the Boston College IACUC under protocol 2019-007-01 to Dr. Tim van Opijnen, PhD, and approved under St. Jude Children's Research Hospital IACUC approved protocol #538 to Dr. Jason Rosch.

Note that full information on the approval of the study protocol must also be provided in the manuscript.
